# *Staphylococcus aureus* Vaccine Research and Development: The Past, Present and Future, Including Novel Therapeutic Strategies

**DOI:** 10.3389/fimmu.2021.705360

**Published:** 2021-07-07

**Authors:** Jonah Clegg, Elisabetta Soldaini, Rachel M. McLoughlin, Stephen Rittenhouse, Fabio Bagnoli, Sanjay Phogat

**Affiliations:** ^1^ GSK, Siena, Italy; ^2^ Host Pathogen Interactions Group, School of Biochemistry and Immunology, Trinity Biomedical Sciences Institute, Trinity College Dublin, Dublin, Ireland; ^3^ GSK, Collegeville, PA, United States

**Keywords:** *Staphylococcus aureus*, vaccinology, host-pathogen interactions, humoral immunity, models of infection

## Abstract

*Staphylococcus aureus* is one of the most important human pathogens worldwide. Its high antibiotic resistance profile reinforces the need for new interventions like vaccines in addition to new antibiotics. Vaccine development efforts against *S. aureus* have failed so far however, the findings from these human clinical and non-clinical studies provide potential insight for such failures. Currently, research is focusing on identifying novel vaccine formulations able to elicit potent humoral and cellular immune responses. Translational science studies are attempting to discover correlates of protection using animal models as well as *in vitro* and *ex vivo* models assessing efficacy of vaccine candidates. Several new vaccine candidates are being tested in human clinical trials in a variety of target populations. In addition to vaccines, bacteriophages, monoclonal antibodies, centyrins and new classes of antibiotics are being developed. Some of these have been tested in humans with encouraging results. The complexity of the diseases and the range of the target populations affected by this pathogen will require a multipronged approach using different interventions, which will be discussed in this review.

## Introduction

*Staphylococcus aureus* is a gram-positive bacterium responsible for significant morbidity and mortality worldwide. In the United States of America, *S. aureus* is estimated to cause 20,000 deaths and amount to a total bill of $15 billion on the health service annually (1, 2). *S. aureus* can be a highly lethal pathogen with a mortality rate during bacteremia of approximately 18% in developed countries (1, 3, 4). This rate has been seen to increase in developing countries, establishing *S. aureus* as a global pathogen (5, 6). One of the most striking and challenging aspects of *S. aureus* clinical management is the ability of the bacterium to develop resistance to treatment with antibiotics. This effect was exemplified during the emergence of methicillin resistant *S. aureus* (MRSA) during the 1960’s and more recently with strains displaying moderate, and in very rare cases, complete resistance to vancomycin, one of the remaining treatment options for MRSA infection (7). Alternative therapies for *S. aureus* are therefore considered an urgent public need. Immunotherapies represent an attractive option due to the reduced likelihood for the development of resistance due to the multifaceted nature of the human immune system. The last two decades have seen considerable effort by the scientific community to develop a vaccine preventing *S. aureus* infection and yet, no vaccine candidates have proven successful at this objective during clinical testing.

*S. aureus* vaccine development has seen laudable innovation. Ever-increasing diversity in vaccine platforms is being observed as viewed through the wide array of antigen selection and the use of novel adjuvants and delivery systems aimed at harnessing specific humoral and cellular immunity. While data explaining the past failure of vaccines continues to emerge, it is imperative that this information is analyzed and reflected upon appropriately to maximize the likelihood of success when developing future vaccines. One of the most important factors that has held back the development of a vaccine is the lack of successful translation of vaccine protectivity that is observed in preclinical models of infection, to protective efficacy seen in human subjects. Here, we propose that the usage of more relevant animal models, more representative *in vitro* models and *ex vivo* human tissues to study the pathogenicity of *S. aureus* will increase the fidelity of data obtained at the preclinical level and therefore increase the likelihood of vaccines entering into clinical trials being efficacious. In addition to ongoing activities related to the development of a vaccine against *S. aureus* at the levels of vaccine design and preclinical testing, we discuss vaccines currently enrolled in clinical trials and alternative therapies for the treatment of *S. aureus* infection. We aim to provide this information while using evidence from past failings regarding *S. aureus* vaccine design, as well as lessons learned from non-*S. aureus* vaccine research, to provide a critical discussion of current research activities in order to pave the way for future research efforts in the field.

**Figure 1:**
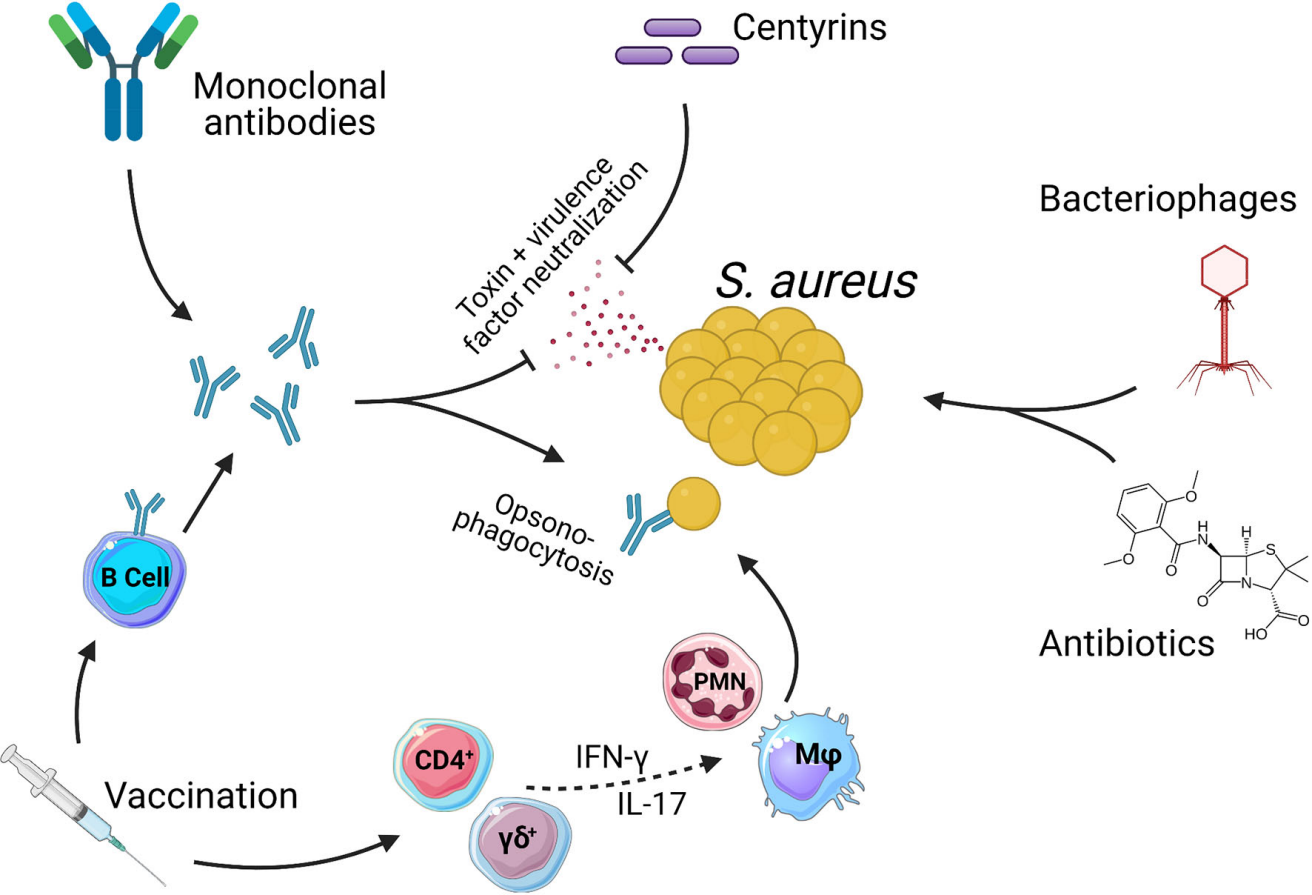
Methods of targeting *Staphylococcus aureus*. There are numerous therapies being investigated as treatments for *S. aureus* infections. Antibiotics are currently the only option with clinical approval. Antibiotics act directly on bacteria *via* either bacteriostatic or bactericidal mechanisms. Bacteriophages also act directly on *S. aureus* to kill bacteria. Centyrins are small proteins with the ability to neutralize bacterial exo-toxins and virulence factors. Monoclonal antibodies can act in the same manner as centyrins to neutralize bacterial exo-toxins and virulence factors, with a secondary mechanism of opsonizing the bacteria due the presence of antibody Fc regions. Vaccines generate populations of memory B and T cells. B cells produce antibodies that act in the same manner as described for monoclonal antibodies. Memory T cells can also be induced which, through pro-inflammatory cytokines such as IFN-γ and IL-17, aid in the activation and recruitment of innate effector cells such as Macrophage (Mφ) and Neutrophils (PMN) which in turn kill bacteria. While CD4^+^ T cells have a more classical role in adaptive memory responses, there is growing evidence to suggest that γδ T cells may be induced by vaccination and play a protective role during *S. aureus* infections (10–13).

To further understand the challenges involved in creating an efficacious vaccine, we also give consideration to certain complexities of the host pathogen relationship between humans and *S. aureus*. Aside from being a major human pathogen with multiple virulence factors specifically focused on disarming key components of the immune system, *S. aureus* also establishes colonizing interactions which in turn results in most, if not all, individuals harboring pre-existing immunity (8, 9). A requirement for an efficacious vaccine will therefore be to improve upon natural immunity and so, provide protection from infection. In parallel to vaccine development, the emergence of novel therapeutic and short-term prophylactic treatments for *S. aureus* disease means that there are now numerous different strategies under investigation for the targeting of *S. aureus* (**Figure 1**). The revitalization of strategies such as bacteriophage therapy, monoclonal antibody treatment and antibiotics as well as the development of new therapeutic proteins such as centyrins, represent exciting experimental treatments for *S. aureus*.

## Vaccine Development Technologies

### Recombinant Proteins and Glycoconjugation

The most common method employed during clinical trials of *S. aureus* vaccines has been the use of recombinant proteins or polysaccharide antigens of the bacterium to evoke a specific immune response in vaccine recipients. The choice of which antigen or combination of antigens to use is a question of paramount importance. Protein antigens typically considered for developing vaccines, are surface antigens or secreted toxins, for the following main reasons: 1) both can be detected by the immune response; 2) antibodies against surface antigens have the potential to induce opsonophagocytosis and block virulence functions (i.e., adhesion and nutrient uptake); 3) antibodies against secreted toxins can block toxicity. *S. aureus* possesses an extracellular polysaccharide coating which has been the target of multiple vaccine formulations entering into clinical trials. Following from the success of licensed vaccines against the bacterial pathogens: *Streptococcus pneumoniae*, *Haemophilus influenzae B* and *Neisseria meningitidis* which used protein glycoconjugation in order to generate strong, long-lived, T cell-dependent B cell immunity towards polysaccharide antigens, this strategy is also being applied to *S. aureus* vaccines (14). As opposed to the classical approach of chemical conjugation, bioconjugation is a more novel method of linking protein and polysaccharide antigens using genetically engineered bacteria to produce these conjugates (15). This technique was used to create an experimental vaccine for *S. aureus* in which the two most common capsular serotypes in clinical settings, CP5 and CP8 (16, 17), were conjugated to exoprotein A of *Pseudomonas aeruginosa* and importantly, a vaccine consisting of CP5 successfully conjugated to the *S. aureus* protein α-toxin (Hla) was also generated (18). The conjugate containing two staphylococcal antigens proved to be the most immunogenic formulation in murine models of *S. aureus* bacteremia and pneumonia, therefore confirming two important principles for *S. aureus* vaccine development: 1) that “designer” glycoconjugates containing antigens from the same microbe are feasible and 2) that including carrier proteins from *S. aureus* increases vaccine immunogenicity. Considering that two of the most high-profile failures of prior *S. aureus* vaccines (Nabi’s StaphVax and Pfizer’s SA4Ag) used chemical conjugation to combine capsular polysaccharides to carrier proteins of unrelated bacteria, bioconjugation to native bacterial proteins may represent an area in which vaccine efficacy can be improved upon (19, 20). This point is further validated by the finding that an abundance of disease-causing *S. aureus* strains do not express a capsular polysaccharide (21).

### Extracellular Vesicles

Gram-negative bacteria naturally secrete outer membrane vesicles (OMVs) composed of membrane-encapsulated periplasmic material. Being native secretions from bacteria, OMVs contain large amounts of pathogen associated molecular patterns (PAMPs) such as bacterial lipopolysaccharide, which activate the innate immune system and display highly effective adjuvant properties (22, 23). Such inherent immunogenicity combined with a relative ease of production and the ability to incorporate non-native proteins has placed OMVs as an emerging and exciting vaccine delivery platform (24). This potential is being realized as we see vaccines using OMVs produced by genetically modified bacteria to increase antigenic yield and to reduce the toxic effects of endogenous Lipid A (known as Generalized Modules of Membrane Antigens or GMMAs) enter into clinical testing (25, 26). While *S. aureus* is a gram-positive bacterium, the applicability of OMV-based vaccination was recently demonstrated using *Escherichia coli* transfected with plasmids encoding five staphylococcal antigens, each fused to the leader sequence of the endogenous lipoprotein LPP, in order to traffic antigens into OMVs. Purified OMVs were used to successfully immunize mice against *S. aureus* sepsis, kidney abscess and skin infection (27). Interestingly, *E. coli* derived OMVs containing no staphylococcal antigens also provided strong protection against *S. aureus* sepsis and kidney abscess models. Such a finding may be a result of the short time frame between final immunization and infection (10 – 14 days) whereby residual immune potentiators induced through OMV immunization mediate anti-staphylococcal defense or, suggest that OMVs themselves induce non-specific protective immunity, an effect known as innate immune memory or trained immunity that has previously shown to play a protective role during murine models of *S. aureus* infection (28–31).

As an alternative to using OMVs which are by definition derived from gram-negative bacteria, the discovery that gram-positive bacteria, including *S. aureus*, secrete extracellular vesicles (EVs) has led to their experimental use as a novel vaccination platform (32, 33). Proteomic analysis has revealed the composition of such EVs to be large and functionally heterogenous with cytoplasmic, secreted and cell membrane proteins with roles in cellular homeostasis, immune evasion and antibiotic resistance (32, 34). Similar to OMVs, *S. aureus* EVs display inherent adjuvant qualities, shown to drive the production of innate proinflammatory cytokines such as TNF-α, IL-6 and IL-12 in dendritic cells and dermal fibroblasts (35, 36). As such, EVs are expected to be highly capable of acting as standalone vaccine platforms. Indeed, during murine models of infection, mice immunized three times with *S. aureus* EVs demonstrated protective immunity towards subsequent *S. aureus*-induced pneumonia one week after receiving final vaccination and crucially, immunity was also demonstrated during a lethal model of *S. aureus*-induced sepsis 40 days after final vaccination, confirming the induction of long-term protective immunity (35). Interestingly, this immunity could be largely transferred *via* T cells and was completely abrogated upon the genetic deletion of IFN-γ. This finding echoes previous studies demonstrating T cell-derived IFN-γ as a crucial mediator of protection during systemic *S. aureus* infections in humans and mice (37, 38). Interestingly, exosomes released by mammalian cells have recently been shown to exert a protective effect against *S. aureus* and other bacteria acting as decoys against Hla and other bacterial toxins (39).

### Whole Cell and Live-Attenuated *S. aureus* Vaccination

Using chemically or physically inactivated or live-attenuated bacteria as a vaccine platform presents up and down-sides. Using whole cell bacteria ensures that a wide array of antigens are present to which a recipient may induce an immune response however, such vaccine formulations may also be associated with significant adverse events when compared to subunit vaccination, best exemplified in such vaccines against *Bordetella pertussis* (40). Furthermore, although live-attenuated vaccines possess an extremely low risk of mutating back into a virulent form, there are exceptions as is seen with the current vaccine-derived circulating strains of Poliovirus in Africa (41). Recently, an attenuated auxotrophic mutant of *S. aureus* MRSA strain 132 was created through genetic disruption of the D-alanine biosynthesis pathway, known to be essential for cell wall structure and viability across different bacterial phyla (42–45). While lethal at high doses (1.1 x 10^9^ CFU), the auxotrophic strain of *S. aureus* was shown to be well tolerated by mice when introduced intraperitoneally at doses of 7 x 10^7^ CFU and lower (46). Furthermore, whether introduced intravenously or intraperitoneally, the auxotrophic strain was cleared within two days of administration. Excitingly, vaccination with this strain proved to be highly immunogenic protecting mice from lethal infection with *S. aureus* and further generating cross-reactive antibodies against a variety of *S. aureus* strains.

Inactivated whole cell vaccines have seen success in preclinical studies using both mouse and bovine models of *S. aureus* infection (47–49). In fact, there are currently two whole cell *S. aureus* vaccines (Lysigin^®^ and Startvac^®^) approved for use in preventing and reducing the severity of bovine mastitis. Notably, a study investigating the protective capability of Startvac^®^ demonstrated a strong inverse correlation (r = -0.71) between vaccine-specific IgG2 responses directed towards a polysaccharide antigen (known as *slime associated antigenic complex*) and survival of *S. aureus* during an infectious challenge (50). Considering that here the antigen in question is structural and not a *S. aureus* toxin or immune evasion protein, it is possible to extrapolate that humoral immunity either opsonizing bacteria or reducing bacterial attachment is important for *S. aureus* clearance during bovine mastitis. Aside from acting as an alternate model of infection, an important value of veterinarian vaccines for *S. aureus* such as Lysigin^®^ and Startvac^®^ is the possibility to examine efficacy of diverse vaccine systems in settings where infections can occur naturally, thereby better mimicking the situation in humans. At this point however, data showcasing prevention of infection or indeed pronounced reductions in severity of disease are lacking (51). As such, meaningful translatability of protection to human subjects in these vaccine systems is not readily indicated. In humans there has been reported success for whole-cell vaccines during early phase clinical trials of i) SA75 containing chloroform inactivated *S. aureus* and ii) heat-killed (HK) *S. aureus* of strain ATCC 12598 (52). While published data for the SA75 trial is lacking, the phase one trial was reported a success and similarly, within the trial of HK-ATCC 12598, no safety concerns were raised by patients. Nonetheless, these vaccines were not further developed. As mentioned above, development of such vaccines presents several limitations for reaching today’s quality standards and that is why vaccine manufactures usually prefer newer technologies. Furthermore, both whole cell and live-attenuated vaccines are difficult to characterize as a reproducible product.

### Nucleic Acid Vaccines

Nucleic acid vaccines deliver antigens encoded either as DNA or messenger RNA to cells which then transcribe and/or translate vaccine antigens into proteins. While until very recently no nucleic acid vaccines had been approved for human use, extremely promising RNA-based vaccine candidates against severe acute respiratory syndrome coronavirus 2 (SARS-CoV-2) rapidly progressed through clinical testing and, as of the time of writing this review (June 2021), two have been approved for human use (53, 54). The rapid progress made by both vaccines using an RNA vaccine platform has brought a renewal of interest into this vaccine strategy. To our knowledge, RNA-based vaccination has not yet been directly investigated with *S. aureus* in humans. One reason for this may be that (although there is long-standing evidence demonstrating the cross-presentation of endogenously derived antigens to class II MHC molecules) endogenously derived antigen is typically associated with presentation on class I MHC molecules, therefore favoring the generation of anti-viral immune responses such as CD8^+^ T cells (55, 56). In line with this, when pulsed with mRNAs encoding the *S. aureus* antigens SpA, MecA and SitC, healthy human monocyte-derived dendritic cells appeared to stimulate antigen-specific cytokine production in donor-matched CD8^+^ T cells to a greater degree than that of CD4^+^ T cells (57). Considering that intracellular survival of *S. aureus* in various cell types has been widely reported, CD8^+^ T cells may represent an understudied component of protective immunity during infection (58–60). mRNA based vaccination against group A and group B Streptococci has been shown to drive protective immune responses in mice, even providing transgenerational, humoral protection (61). As such, mRNA vaccination may well prove a viable strategy for immunization against gram-positive bacteria such as *S. aureus*. Another point of interest in this study from Maruggi and colleagues, was that the inclusion of a leader sequence targeting the group B Streptococcus protein BP-2a for cell secretion increased vaccine immunogenicity. Specifically, this relocalization of BP-2a led to greater survival rates amongst the pups of immunized mice when challenged with a lethal infection of *Streptococcus agalactiae*, an effect that was most apparent when a combined vaccine regime using priming RNA doses and a booster dose with recombinant protein was employed (61).

DNA vaccines consisting of plasmid encoded antigens are another intensely studied nucleic acid-based vaccine platform that has previously shown efficacy against *S. aureus* in preclinical studies during murine models of infection (62, 63). Like RNA-based vaccination, DNA vaccines carry with them the ability to prime CD8^+^ T Cells responses (62). However, a major obstacle that has obstructed the development of an efficacious DNA-based vaccine to date is the low immunogenicity observed in human subjects (64).

### Adjuvants

Adjuvants are used to boost the immunogenicity of otherwise poorly immunogenic antigens. The majority of previously discussed vaccine platforms are considered self-adjuvanted due to the presence of PAMPs in the case of OMV-based and whole cell vaccines or due to the immunostimulatory nature of exogenously derived nucleic acids [though adjuvants have previously been shown to boost immunogenicity in nucleic acid vaccines (65)]. Recombinant proteins and glycoconjugates however, are more often combined with adjuvants as they do not contain any outright activators of the innate immune system and are therefore generally less immunogenic. In previous clinical trials for *S. aureus*, recombinant proteins and glycoconjugates have been used both with and without adjuvants. Two separate vaccine candidates, V710 and StaphVAX, which both failed during phase 3 clinical efficacy testing were unadjuvanted. Merck’s V710 vaccine contained Iron Surface Determinant B (IsdB) while Nabi’s StaphVAX contained CP5/CP8 capsular polysaccharides conjugated to *P. aeruginosa* exoprotein A (19, 66–68). In the case of StaphVax, efficacy in reducing *S. aureus* bacteremia was evident at an estimated level of efficacy of 57% 40 weeks post-vaccination, however, by 54 weeks, efficacy drastically dropped to 26% (19). Vaccination with V710 showed minimal efficacy in reducing the onset of surgical site infection with *S. aureus* and actually increased the rate of mortality in those developing post-operative *S. aureus* infections (69). Mechanistically, this increased mortality has been previously linked to low endogenous levels of IL-2 and IL-17 in patients prior to receiving the vaccine (66). More recently, a novel mechanism explaining such worse outcomes was put forward by Nishitani and colleagues whereby at sites of surgical infection with *S. aureus*, antibodies against IsdB were shown to be captured in reverse orientation by the cell surface protein SpA. Here, the exposed Fab region of such antibodies may bind their target protein, IsdB which in turn binds its ligand: Hemoglobin-Haptoglobin complexes (70). Finally, Hemoglobin-Haptoglobin complexes are recognized and internalized by macrophages through the expression of CD163 which, due to the aforementioned ability of *S. aureus* to survive intracellularly, facilitates a behavior known as the Trojan horse hypothesis, whereby dissemination of *S. aureus* throughout the body occurs *via* macrophage migration within the blood supply (71, 72).

One possible explanation for these failures may be the lack of an adjuvant employed in both vaccines. Evidence for this hypothesis comes primarily from preclinical studies using murine models and demonstrating enhanced immunogenicity in *S. aureus* vaccine formulations after the inclusion of an adjuvant or demonstrating differential immune profiles in mice vaccinated with identical antigens using altered adjuvant systems. In fact, antigen-specific antibody titers (73–76), antigen-specific T cell responses (74, 76) as well as bacterial clearance and survival during subsequent infection (73–77) were all shown to increase with the inclusion of an adjuvant or to be significantly altered when comparing the use of different adjuvants, confirming the important impact an adjuvant may have on *S. aureus* vaccine efficacy. It should be noted however, that a recent study investigating the efficacy of various antigen combinations in murine models of skin and systemic *S. aureus* infections found no demonstrable differences in efficacy between a variety of adjuvants (78). Considering that in this study, no unadjuvanted control groups were included, and further that skin-infection was essentially attenuated by all vaccine formulations, meaningful conclusions are difficult to draw.

Although human data supporting a role for adjuvants in *S. aureus* vaccination is sparse, a phase 1 clinical trial for a *S. aureus* vaccine containing recombinant Hla, clumping factor a (ClfA) and CP5/CP8 conjugated to tetanus toxoid, the adjuvant system AS03 was investigated. AS03 is an oil-in-water emulsion shown in previous clinical trials to significantly increase the immunogenicity of influenza vaccination (79, 80). The non-adjuvanted vaccine induced a rapid and potent antibody response towards the vaccine antigens suggesting that the vaccine induced a memory response, which pre-existed prior to vaccination. This phenomenon can be explained by the fact that humans are virtually all exposed to *S. aureus* and indeed the presence of antibodies against its antigens in healthy subjects has been documented (81). The effect for AS03 in boosting the *S. aureus* vaccine immunogenicity appeared not statistically superior as compared to the non-adjuvanted vaccine (82). The reason behind this may rely on the effect of pre-existing immunity, which makes antibody titers increase even without an adjuvant. In those conditions, it is likely that the quality, more than the quantity, of the antibodies can be improved in the presence of a proper adjuvant. However, it is difficult to derive any conclusions from the available clinical data on this hypothesis. Continuing with this idea, these findings open the door for a broader discussion on exactly what type of adjuvant should be considered for *S. aureus* vaccines.

Aside from the aforementioned use of AS03, all other *S. aureus* vaccines trialed in humans have used aluminum-based adjuvants. While yet to be fully understood, the mechanism through which alum-based adjuvants enhance the immunogenicity of vaccines is believed to be through a combination of antigen retention at the site of injection and local activation of the NLRP3 inflammasome to promote innate cytokine secretion (83, 84). Alum has been shown to specifically polarize T cells towards Th2 and Tfh subsets, both associated with protection from extracellular parasites and the generation of humoral immunity (85–88). While the correlates of immunity to *S. aureus* infection are not definitively established, a role is appreciated for that of cellular immunity, specifically Th1 and Th17 mediated immunity, as evidenced by genetic, clinical and experimental data (38, 89–91). Importantly, these findings help to demarcate a second reason as to why adjuvant selection is important in *S. aureus* vaccine design and why alternatives to alum may be required: directionality of the induced immune response. Like AS03, MF59 is an oil-in-water emulsion that is used in licensed influenza vaccines (92). In a preclinical study investigating the immunogenicity of a multi-component *S. aureus* vaccine containing FhuD2, Csa1A, Hla, EsxA, and EsxB, a direct comparison was conducted between alum and MF59-adjuvanted vaccines (74). When given the MF59-adjuvanted vaccine, mice developed moderately enhanced antigen-specific memory T cell responses, demonstrated by CD4^+^ T cell proliferation and cytokine production, one month post-vaccination compared to that of mice receiving the alum-adjuvanted vaccine, however this effect was not apparent at the four month time-point. Furthermore, evidence of greater protection afforded by the vaccine including MF59 was not wholly apparent when compared to the alum vaccine group. As such, while hinting at greater induction of T cell responses, MF59 does not appear to present itself as a clearly more efficacious adjuvant than alum in generating protective *S. aureus* immunity in a murine model.

Using the same experimental vaccine, a separate study found that inclusion of a TLR7 activating small molecule immune potentiator adsorbed onto alum could significantly enhance protective immunity in vaccinated mice versus control mice receiving a vaccine adjuvanted with alum alone (75). T cell responses were also greatly increased when compared to the alum only group, with enhancement of antigen-specific production of IL-2, TNF-α, IFN-γ and a trend towards an increase of IL-17 observed. Notably, no increase in Th2 cytokines was observed, suggesting that the directionality of the induced immune response was specifically focused (75). Indeed, further analysis confirmed the induction of Th17 immunity as well as Th1 immunity, and further found that vaccine-induced protective immunity was highly dependent on both humoral and CD4^+^ T cell responses (76). These encouraging results suggest that adjuvants acting through TLR7 may prove useful for the development of *S. aureus* vaccines.

Another TLR-activating adjuvant is CpG, composed of unmethylated cytosine and guanine repeats. When used as an adjuvant, CpG has been shown previously to evoke Th1 humoral and cellular responses in both mice and humans (93, 94). This Th1 biasing effect was harnessed in two separate studies to successfully develop protective vaccination regimes in mice against localized and systemic infections with *S. aureus* (37, 95, 96). It should be noted that, in addition to Th1 responses, CpG-mediated induction of antigen-specific humoral and Th17 immunity was also observed and correlated with protection from infection (95, 96). As such, the activity of CpG through TLR9 may represent a promising pathway for the induction of humoral and polyfunctional T cell responses associated with protection from *S. aureus* infections.

To summarize, the diversity observed in preclinical testing relating to vaccination strategies and choice of adjuvant is highly encouraging. While current research aims to elucidate the correlates of protection to *S. aureus* infection and to clinically validate the selection of particular antigens, diversity will be key to ensuring success in *S. aureus* vaccine design.

## Models for the Study of *S. aureus* Infections

The conversation of *S. aureus* vaccine development is framed by past failings of clinical trials that all showed exciting promise during preclinical development. As such, we can conclude that the translation of preclinical data into clinical results is not successfully occurring. It’s clear that more accurate preclinical models to study *S. aureus* infections and the human immune response to such infections are needed to improve the predictability of laboratory-generated vaccines in protecting human subjects. In addition, determining the correlates of protection of *S. aureus* infections requires faithful recapitulation of human tissues and the human immune system. The most utilized model for studying *S. aureus* infections is the mouse. On one hand, the gene expression profiles of *S. aureus* extracted from natural cutaneous infections of human patients or from the kidneys of mice with experimentally induced systemic infections have been shown to be similar (97). However, the ability of mice, and in particular laboratory mice, being able to recapitulate the human immune response to infection has been under scrutiny in recent years (98, 99). While this is undoubtedly true in a generalized immunological sense, *S. aureus*-specific examples of this effect can also be observed through the reduced affinity of staphylococcal toxins for non-human homologs of toxin receptors. For example, the bicomponent toxins: Panton-Valentine leukocidin (PVL), LukAB, HlgCB and HlgAB all display reduced affinity towards murine homologs of each of their human receptors, meaning that virulence mediated by these proteins may be missed through the use of mice (100, 101). This effect has been demonstrated through the infection of immunodeficient mice re-constituted with human hematopoietic stem cells, thereby classified as humanized mice. Studies exposing humanized mice to a variety of *S. aureus* infections, including pneumonia, skin infection and intraperitoneal infection consistently showed increased susceptibility compared to wild-type or immunodeficient controls (102–104). In one such study, the efficacy of successful humanization as measured by the ratio of human to murine CD45^+^ cells was directly correlated with the size of skin abscesses in mice and skin lesions were significantly reduced in humanized mice when a PVL-deficient strain of *S. aureus* was used, an effect not observed in wild-type mice (102). These findings combine to suggest that *S. aureus* host-specificity is a crucial factor for the outcome of infection and that humanization of mice may be a highly useful tool to increase the translatability of preclinical vaccine data (105). Notably, rabbits, while still much less than that of humans, are more susceptible to the effect of many *S. aureus* toxins, therefore suggesting rabbits as a more relevant *in vivo* model (106). *S. aureus* vaccines and monoclonal antibodies have also occasionally been tested in non-human primates, generating highly relevant data (107, 108).

An additional caution to be noted when using mouse models for the study of *S. aureus* pathology is that specific pathogen free mice purchased from commercial vendors, as well as laboratory housed mice, may be naturally colonized with mouse-adapted *S. aureus* (109–111). Considering that pre-exposure to *S. aureus* in the contexts of colonization and infection alter the host immune response to subsequent infection, as reported in both mice and humans (112–114), the potentiality that experimental mice may or not be pre-colonized introduces a confounding factor into preclinical research. From an alternative standpoint, the evidence of host adaptation occurring in mice colonized with *S. aureus* may actually give value to the mouse model *via* two mechanisms. Firstly, the opportunity to study previously reported, naturally developing *S. aureus* infections in mice would mirror the process of human infection much more closely than experimentally induced infections (110). Secondly, though mouse-derived strains of *S. aureus* will be less clinically relevant than human-derived strains, host-adaptation may allow for more accurate comparative immunology approaches to be undertaken when studying the mouse immune response to *S. aureus*.

An interesting alternative animal model for studying *S. aureus* pathology that has been mobilized in recent years is the Zebrafish. The benefits of using this more distantly related organism include: i) the ability to study innate immune responses before the adaptive immune system has developed (this only occurs four weeks after fertilization), ii) a high degree of evolutionary divergence between human and Zebrafish immune systems allows us to specifically examine the functionality of highly conserved genomic material, thereby reducing the complexity of the system in question and iii) advanced microscopy techniques are applicable to this model due to its transparent appearance (115). Indeed, while utilized significantly less than the mouse model, some important basic research findings concerning the pathogenicity of *S. aureus* as well as inferences of protective immunity were made using the Zebrafish model (116, 117).

The development of *in vitro* models to study human immunology is crucial towards moving away from the biologically and ethically undesirable use of animal models. Two leading approaches for recapitulating the physiological environment of human organs are: organoids and organs on chips. Organoids are cell culture models containing heterogenous cell types and anatomical organization that mimic a particular organ (118). In the context of *S. aureus*, the skin, lungs and bones represent some of the most common biological niches of non-systemic infection in humans. Therefore, establishing systems that model these organs *in vitro* could be relevant for the investigation of host immunity during infection. Recently, a human skin organoid was developed from human pluripotent stem cells that displayed complex architecture, hair production and a transcriptome all faithfully recapitulating that of human facial skin (119). Further still, cultured skin could be xenografted and integrated into a mouse model, therefore adding a significant degree of humanization for *in vivo* analysis of a hypothetical *S. aureus* infection. Similarly, the development of lung and bone organoid cultures has become more refined in recent years (120–122). Drawbacks to using organoids in *S. aureus* vaccinology research include i) the complexity of natural human tissue and the difficulty in fully re-creating such complexity *in vitro* ii) the inability to address the contribution of a tissue’s microbiome iii) the lack of potentially important cell types, for example tissue-resident lymphocyte populations which are abundantly present in many organs and finally iv) a lack of interaction with a systemic immune system when used in isolation (123, 124).

Organs-on-chips consist of micro-scale organs, derived either from cell culture methods akin to that of an organoid or alternatively from *ex vivo* samples of an organ, often connected to a microfluidics system which mimics that of a physiologically relevant vascular system (125). The applicability of this model in studying S. *aureus* infections was recently demonstrated by Kim and colleagues. In this study, a micro-biopsy of skin was taken from healthy human subjects, infected with *S. aureus* and then loaded into a chip containing two chambers: one for the skin explant and another for the addition of one drop of whole blood. Importantly, blood and skin were separated by columns selectively allowing for the autonomous migration of neutrophils in response to chemotactic signals secreted by the skin (126). As such, it may be possible to obtain minimally invasive micro-samples of skin and blood from humans enrolled in clinical trials receiving *S. aureus* vaccines to determine whether skin-derived and/or systemic immunity raised by a vaccine may contribute to physiologically relevant protection from *S. aureus* infection, using this model. A lung organ-on-chip was also recently developed and investigated for its ability to study *S. aureus* infections. Here Deinhardt-Emmer et al. (127) used three cell lines to establish an artificial alveolus consisting of endothelial, epithelial and macrophage cells that was then infected with both *S. aureus* and influenza virus (127). The alveolus-on-chip was connected to a fluidics system consisting of a peristaltic pump ensuring unidirectional flow and a reservoir of cell culture medium, mimicking a circulatory system.

A highly valuable model to study the infective behavior of *S. aureus* are human *ex vivo* samples. *Ex vivo* samples are derived directly from human subjects and consist of complex tissues influenced by a natural microbiome, containing diverse genetic makeups and fully relevant cell populations. When considering *S. aureus* infections, there are some human tissues/fluids highly relevant to the study of host-pathogen interactions, that are also accessible for *ex vivo* analysis. For example, the transcriptome of *S. aureus* cultured *ex vivo* within human blood is distinct from that of the bacterium cultured in tryptic soy broth *in vitro*, also found with *S. aureus* derived from human sputum in cystic fibrosis patients (128, 129). The skin is another tissue of great relevance and opportunity for the study of *S. aureus* infectivity [as reviewed recently (130)]. Skin can be accessed through the donation of surgical waste samples, micro-biopsies and may even be probed *in-situ*, as demonstrated recently through the development of microneedle patches designed to extract interstitial fluid as well as antigen-specific lymphocytes from human skin (131). The cytokine response to *S. aureus* infection as well as the toxicity of Hla and PVL have previously been investigated using skin explant models, generating physiologically valuable data and a launchpad for future *ex vivo* analysis (132, 133). Recently, the presence of *S. aureus*-specific tissue-resident CD4^+^ T cells in the skin of healthy subjects has been shown using abdominal skin explants (134). The protective efficacy of this frontline adaptive immune response could be harnessed by future vaccination strategies (135).

In summary, prior rates of failure demand alternatives to standard mouse models when investigating the protective capacity of experimental *S. aureus* vaccines and to determine the correlates of immunity to infection. While alternate animal models such as the zebrafish can help to elucidate certain aspects of *S. aureus* behavior, the further development and use of humanized mouse models or novel *in vitro* models such as organoids, organs-on-chips and *ex vivo* tissue culture will be critical in increasing the clinical translatability of laboratory generated data.

## An Update Regarding Ongoing and Recently Concluded Clinical Trials for *S. aureus* Vaccines

There have recently been some key developments in the landscape of *S. aureus* vaccine development (**Table 1**). The most noteworthy of these is the phase IIb failure of Pfizer’s SA4Ag vaccine candidate. Composed of recombinant MntC, ClfA and both CP5 and CP8, each conjugated to a detoxified form of diphtheria toxin, this vaccine was tested in four phase I clinical trials (20, 139–141) before ultimately failing to reach designated protection endpoints in a subsequent efficacy trial (NCT02388165). In phase I trials, a single dose of vaccine was sufficient to elicit high titers of specific and functional antibody responses in recipients from age 18–80 shown to last for at least a year after vaccination. Functionality was determined through antibody-mediated opsonophagocytic killing of bacteria and through inhibition of ClfA-mediated binding to its ligand, fibrinogen. Although SA4Ag was therefore deemed to be highly immunogenic, it failed to cause any reduction in the incidence of *S. aureus* bloodstream infections, surgical site infections or all-cause mortality within periods of 90 and 180 days after recipients underwent spinal surgery (NCT02388165). SA4Ag’s failure in conferring protection to patients carries with it some critical lessons to be understood for *S. aureus* vaccinology. Firstly, and most crucially, the high immunogenicity of SA4Ag, as determined from assays analyzing the sera of vaccinees, had no bearing on its efficacy. One reason for this may be that the assays employed in order to classify the vaccine as immunogenic were not sufficient to capture the components of the immune system associated with preventing *S. aureus* infection. This issue stems again from the lack of defined correlates of protection to *S. aureus* infection, making the term “immunogenic” when applied to such vaccines somewhat misleading. In the case of SA4Ag, vaccine-induced humoral immunity was shown to be antigen specific in nature and capable of inducing bacterial opsonophagocytosis. Opsonophagocytic humoral responses were also demonstrated in both V710 and StaphVax vaccines that similarly failed efficacy testing in late-stage clinical trials (19, 69). As such, there is a growing consensus that using opsonophagocytosis as a readout for anti-staphylococcal immunogenicity is not sufficient as a standalone predictor of vaccine efficacy (143). Aligned with this point, none of the vaccine candidates that entered into late stage efficacy trials, reported on vaccine-induced T cell responses in humans, a potentially crucial aspect of immunogenicity when considering immunity from *S. aureus* infections. It is also possible that the *in vitro* assays used for measuring opsonophagocytosis were not predictive of this effect *in vivo*. For example, opsonophagocytosis assays are often performed using neutrophil-like cell lines as opposed to primary human neutrophils (20, 69, 138).

**Table 1:** *Staphylococcus aureus* vaccines currently enrolled in clinical trials.

Company	Vaccine	Phase	Clinical trial number	Study population	Literature
GSK	SA-5Ag: Adjuvanted	I: Recruiting	NCT04420221	18 – 50 year olds at risk of recurrent skin infections	
Novadigm Therapeutics	NDV-3A: Als-3 (*C. albicans* cross reactive cell wall protein) + Alum	II: Ongoing	NCT03455309	Military Personnel	(136, 137)
Olymvax	rFSAV: Hla, SpA, SEB, IsdB, MntC + Alum	II: Ongoing	CTR20181788, NCT03966040		(138)
Pfizer	SA4Ag: CP5-dptx, CP8-dptx, ClfA, MntC	IIb: Failure	NCT02388165	Patients undergoing spinal surgery	(20, 139–141)
Integrated Biotherapeutics	i. Stebvax: SEB + alum ii. IBT-V02: SEB, SEA, TSST-1, LukS, LukF, LukAB, Hla + alum	I: Completed I: Scheduled	NCT00974935	18 – 40 year olds	(142)

Another lesson from SA4Ag’s failure is that while retrospective analysis of Nabi’s StaphVax vaccine seemed to indicate that waning antibody titres might lead to a decreased efficacy overtime (19), here SA4Ag was specifically shown to induce lasting humoral responses for periods of time longer than the efficacy study itself (141). Considering that SA4Ag was given as a single, unadjuvanted dose, it is conceivable that administration of the vaccine acted to simply boost pre-existing *S. aureus* immune responses without significantly enhancing the quality and functionality of humoral or cellular immunity.

There are at least five *S. aureus* vaccines currently engaged in various stages of clinical trials (**Table 1**). Novadigm’s NDV3-A vaccine consists of the N-terminal part of the *Candida albicans* cell-wall protein Als3p adjuvanted with alum. Although a fungal protein, this vaccine provides cross-protection against *S. aureus* during murine models of bacteremia and skin infection due to cross-kingdom antigen overlap (38, 136, 144). Currently this vaccine is in a phase II trial aimed at preventing nasal colonization with *S. aureus* in military personnel, a population in which the frequency of *S. aureus* skin infections is high (NCT03455309). Encouragingly, this vaccine has been associated with strong cellular immune responses in preclinical testing and crucially, was show to elicit antigen-specific production of the T cell cytokines IFN-γ and IL-17 in human recipients during a phase I trial (38, 137). Furthermore, aside from IgG antibody responses, NDV3 was also found to induce a potent IgA response. Typically, non-IgG antibodies are not examined during the study of *S. aureus* immunity, however bovine research has suggested an anti-staphylococcal role for IgA and considering that in humans, *S. aureus* most commonly occupies mucosal niches, determining whether such antibodies can suppress colonization represents an area worth further study (145). Suppressing colonization is a novel clinical endpoint to set for an *S. aureus* vaccine and indeed, a crucial step for disease eradication, while also representing a major risk factor for the development of staphylococcal bacteremia (146, 147). A prior phase I trial by GSK investigating the safety and immunogenicity of a four-component vaccine containing: CP5, CP8, Hla and ClfA was found to have no effect on rates of *S. aureus* carriage over two years (82). This vaccine induced strong IgG responses and weak cellular immune responses in recipients suggesting that, as concluded when discussing vaccine efficacy, prevention of colonization may require more than robust IgG responses alone.

Olymvax have developed a *S. aureus* vaccine named rFSAV currently in phase II trials (CTR20181788). This vaccine is composed of five recombinant *S. aureus* antigens: Hla, SEB, MntC, IsdB and SpA and showed promising efficacy in preclinical murine experiments (138). In addition to promoting opsonophagocytosis, sera from mice immunized with rFSAV were also shown to neutralize the lytic activity of Hla and prevent a slight depletion of splenic B cells observed in mice, mediated by treatment with SpA (138, 148). Importantly, this vaccine represents an alternate aspect of “immunogenicity” against *S. aureus*: inhibition of *S. aureus* immune-evasion strategies. This can be understood through the inclusion of both Hla and SpA in the vaccine formulation. Hla engages in the lytic killing of many leukocyte cell types and further disrupts cellular tight-junctions, facilitating *S. aureus* invasiveness (149, 150). Moreover, Hla intoxication leads to platelet aggregates that are deposited in the liver causing microvascular dysfunction and thrombosis in infected mice (151). In addition, Hla was recently shown to prevent the expansion of T cells during primary murine *S. aureus* skin infection, implicating itself as an important immune-evasion toxin (152). SpA acts both as a B cell superantigen and to sequester antibodies by their Fc portion, therefore functioning to suppress the humoral immune response through two separate mechanisms (153, 154). Lastly, SEB is known to induce rapid expansion, activation and subsequent anergy in a large proportion of the host T cell compartment (155). Considering the previously discussed evidence that anti-IsdB antibodies may in fact be pathological, combined with clinical serological data reporting a similar phenomenon, extremely close observation should be kept on individuals receiving this experimental vaccine to prevent vaccine-induced mortality during *S. aureus* infections as seen in the V710 trial (66, 156).

Integrated BioTherapeutics have developed a heptavalent *S. aureus* vaccine consisting of seven *S. aureus* toxoids: Hla, Panton-Valentine Leukocidin (PVL) F and S subunits, Leukocidin A/B, SEA, SEB and Toxic shock syndrome toxin 1 (157). Preclinical data has shown that this vaccine, named IBT-V02, confers protection to both mice and rabbits against *S. aureus* skin infection, with protection being entirely mediated by vaccine-induced antibodies (142). Interestingly, vaccine efficacy (as measured through the development of infection-induced skin lesions) was unaffected when mice were pre-exposed intradermally to a low dose of *S. aureus* prior to vaccination. As mentioned previously, humans are naturally exposed to *S. aureus* and harbor pre-existing immunity (81). Pre-exposing mice to *S. aureus* therefore represents an encouraging degree of humanization to the mouse model. After receiving significant funding grants from the National Institute of Allergy and Infectious Disease and CARB-X, IBT-V02 is expected to enter early phase clinical trials soon. Integrated BioTherapeutics are also developing a vaccine aimed specifically at neutralizing systemic toxicity induced by SEB which may find use in preventing potential SEB-related biological warfare attacks. This vaccine, consisting of a mutated, non-MHC-II binding recombinant SEB protein successfully completed a phase I trial in 2016 (158). GSK are in the recruiting phase of a phase I trial for their SA-5Ag vaccine (NCT04420221). The target population in this trial is listed as 18-50 year-olds with a recurrent *S. aureus* skin infection, a novel population for *S. aureus* vaccine trials.

## Novel Therapeutic Strategies for the Treatment of *S. aureus* Infections

In parallel with vaccinology research, the development of therapeutic interventions for *S. aureus*-mediated disease is an area of constant innovation. In recent years, the revamping of older, more established treatment options as well as the development of completely novel strategies has created exciting hope for new treatment options against *S. aureus*. Herein, we discuss some of the most innovative and promising therapeutic interventions for the treatment of *S. aureus* infections.

### Novel Antibiotic Strategies

Mortality rates due to *S. aureus* bacteremia are significantly higher in cases of MRSA bacteremia when compared to methicillin susceptible *S. aureus* (MSSA)-mediated disease with one study reporting mortality rates of 49.8% and 22.2% respectively (159). Such a finding is believed to be a result of the lower efficacy of daptomycin and vancomycin, the two first-line recommended interventions for MRSA infection, in treating *S. aureus* when compared to β-lactam antibiotics which are used to treat MSSA (160). Improving on this efficacy is therefore a priority for MRSA treatment, a sentiment captured in a recent statement from the World Health Organization following the completion of two reports investigating the global development of antimicrobial drugs, in which they warn that a lack of development will risk our ability to contain the spread of multi-drug resistant bacteria (161, 162). In early clinical and preclinical studies, novel antibiotics and delivery systems improving the efficacy of established antibiotics are being investigated along with the usage of novel drug combinations. An example of such effects at work can be seen by the recent development of DSTA4637S, an antibody-antibiotic conjugate consisting of a monoclonal antibody targeting *S. aureus* wall teichoic acid, fused to a novel rifamycin-class antibiotic (163). Mechanistically, DSTA4637S works to promote opsonophagocytosis which, upon entry into the intracellular phagolysosome of human phagocytes, initiates the cleavage of the fused antibiotic, and subsequent bacterial inhibition (164). Crucially, and unlike currently administered antibiotics, this mechanism is capable of effectively killing intracellular *S. aureus*, thereby reducing the ability of metastatic infections to occur. DSTA4637S recently completed a phase 1 trial (165).

The potential power of combination antibiotic therapy was recently exemplified in a small study of 40 participants in which daptomycin treatment was combined with the β-lactam ceftaroline. This study showed that combination treatment significantly reduced mortality when compared to either daptomycin or vancomycin treatment alone in patients with MRSA bacteremia (166). Though the mechanism for such synergy is not yet understood, it has been proposed that β-lactam antibiotics may directly enhance the bactericidal capacity of the innate immune system, thereby acting as an adjunct to daptomycin treatment (167).

Within the last three years, an abundance of novel mechanisms aiming to enhance the delivery of anti-staphylococcal medicines towards site-specific infections such as skin and soft-tissue infections, implant related osteomyelitis or pneumonia have been developed in preclinical settings. For example, following the discovery that mesenchymal stem cells display antibacterial (including anti-*S. aureus*) activity (168, 169), Yoshitani and colleagues developed a novel therapeutic treatment in rats, consisting of adipose-derived stem cells loaded with the fluoroquinolone antibiotic ciprofloxacin, administered as a local injection to rats with experimentally-induced implant-related *S. aureus* infections. This strategy was shown to decrease bacterial loads at the site of infection while further showing to outperform standard antibiotic treatment in decreasing osteomyelitis and bacterial abscess formation (170). A relatively more simplistic idea was recently applied for the delivery of hydrogel scaffolds mediating bone repair during a murine model of experimentally induced bone-defects. Here, the hydrogel scaffold was co-delivered with live *S. aureus* in order to model an implant-related infection however, upon the inclusion of the enzyme lysostaphin which displays potent anti-*S. aureus* activity to the scaffold, bacterial presence within implants was negligible at one and six weeks post-introduction of the infected scaffold (171). Focusing on a separate infection site, Hussain et al. (172) developed a *S. aureus*-binding peptide, using a combination of *in vivo* and *in vitro* phage display, and then coated silicon nanoparticles containing vancomycin with this peptide to target antibiotic delivery directly to live bacteria. Importantly, this strategy was shown to completely protect from *S. aureus* pneumonia-induced mortality during a murine model of infection (172). Positively charged silver nanoparticles are broad spectrum biocides that were used recently to functionalize catheter materials, leading to the effective inhibition of single- as well as dual-species *S. aureus*/*C. albicans* biofilm formation (173). Catheter-related bloodstream infections due to biofilm formation are a major healthcare problem and mixed bacterial/fungal biofilms formed by these two species represent a relevant clinical complication since β-1-3-glucan secreted by *C. albicans* provides *S. aureus* with enhanced antibiotic tolerance (174). In summary, an abundance of novel mechanisms involved in pharmacologically increasing the efficacy of antibiotics and in targeting antibiotics directly towards sites of infection are being developed, holding promise for the generation of novel therapeutics to treat *S. aureus*.

### Bacteriophage Therapy: Phages and Endolysins

While not a novel strategy for the treatment of bacterial infections, bacteriophage therapies have seen a revitalization in recent years with numerous promising treatments in development for *S. aureus* infections as well as other notable antibiotic resistant bacteria in both preclinical and clinical stages (161, 175). Two distinct methods by which anti-staphylococcal viruses can be harnessed are through a) the use of virus-derived antibacterial enzymes such as endolysins and b) the injection of full viral particles. An example of a promising phage endolysin is SAL-200, developed by Intron Biotechnology. This *S. aureus-*specific enzyme demonstrated bactericidal activity against over 400 strains of *S. aureus* and was further shown to ameliorate outcomes to murine bacteremia while also synergizing with antibiotic treatment during murine and moth larval systemic infections to deliver greater therapeutic efficacy (176, 177). SAL-200 has since entered into human clinical trials where it was firstly shown to be well tolerated in healthy volunteers and as such, has now entered into phase II testing (178) (NCT03089697, NCT03446053). Earlier this year CF-301, another bacteriophage endolysin, completed a proof-of-concept efficacy trial for the treatment of *S. aureus* bacteremia and endocarditis given as an adjunctive therapy with standard of care antibiotic treatment. Interestingly, though the additive efficacy of endolysin treatment during all *S. aureus* infections was 10% fourteen days post-treatment, restricting analysis to cases of MRSA demonstrated that over 42% of patients responded positively to adjunctive treatment (179). Such a finding is believed to reflect the previously discussed inferiority in anti-bacterial activity associated with antibiotics used during MRSA infections when compared to those used in MSSA infections. The activity of bacteriophage therapy has also been investigated for the treatment of more localized *S. aureus* infections. For example, a phase I trial was recently completed using the AB-SA01 bacteriophage cocktail demonstrating safety and preliminary indications of efficacy in combatting *S. aureus* mediated chronic rhinosinusitis (180). Consisting of three separate phages, this treatment has also recently been utilized in a small cohort of patients with severe systemic *S. aureus* infections lacking any non-treated controls (181). While the discussed data is promising, much remains to be seen concerning the use of bacteriophage therapies for *S. aureus* infections, paramount of which is large-scale efficacy. Some further outstanding issues that remain to be consolidated for bacteriophage therapy include: i) whether pre-existing or treatment-generated antibody responses will inhibit therapeutic efficacy and ii) whether bacteriophages or endolysins will be capable of killing intracellular *S. aureus* (182).

### Monoclonal Antibodies

Passive immunization with monoclonal antibodies is an area of keen interest in the development of *S. aureus* therapeutics. Initially, efficacy trials of both poly and monoclonal antibodies targeting surface antigens of *S. aureus* such as: fibrinogen binding proteins (ClfA and Ser-Asp dipeptide repeat G), lipoteichoic acid and capsular polysaccharides showed largely disappointing results (183–185). More recently however, development has shifted towards monoclonal antibodies targeting staphylococcal toxins and immune evasion proteins as observed by the current antibody therapies engaged in clinical testing (**Table 2**). The furthest therapeutic along this track is the anti-Hla monoclonal antibody Tosatoxumab developed by Aridis, currently recruiting for a phase 3 trial in patients with *S. aureus* ventilator associated pneumonia (VAP) in addition to standard of care treatment (NCT03816956). Tosatoxumab completed a phase 1/2a trial on patients presenting with *S. aureus* pneumonia (including hospital acquired and community acquired pneumonia) showing to be well tolerated in recipients of the antibody. Furthermore, though statistical analysis was restricted to just 25 patients, when specifically looking at patients suffering from VAP, Tosatoxumab was found to significantly reduce the duration of time spent on emergency mechanical ventilation, therefore showing indications of efficacy in reducing *S. aureus*-mediated disease (188). As such, larger scale data in this patient cohort is expected to reveal a clearer picture as to whether neutralization of alpha-toxin is indeed associated with effective treatment of *S. aureus* VAP. Another monoclonal antibody neutralizing the lytic activity of Hla, Suvratoxumab developed by AstraZeneca, has been developed to have an extended half-life within human blood and again has shown to be well tolerated within recipients (191, 192). Results of a phase II trial indicate that in patients receiving mechanical ventilation that are pre-colonized with *S. aureus*, Suvratoxumab displays some efficacy in reducing the development of *S. aureus* VAP, however the magnitude of efficacy at 31.9% was not sufficient to meet the efficacy endpoint of the study and furthermore did not reach statistical significance (189). In summation, though some encouraging results have been observed in the neutralization of Hla as both a prophylactic and therapeutic intervention in *S. aureus* VAP, further studies, particularly with larger sample sizes, are needed to provide a clear result demonstrating efficacy or a lack thereof.

**Table 2:** Therapeutic treatments for *Staphylococcus aureus* infections currently enrolled in clinical trials.

Company	Medicine	Phase	Clinical trial number	Literature
Cumberland Pharmaceuticals	**Televancin:** Vancomycin derivative	III failure	NCT02208063	(186)
Arsanis	**ASN-100:** Two monoclonal antibodies against Hla, PVL, gamma-hemolysin (HlgAB and HlgCB), LukED and LukGH	II Failure	NCT02940626	(187)
Genentech	**DSTA4637S:** Monoclonal antibody-antibiotic fusion targeting wall-teichoic acid	I	NCT02596399	(165)
iNtRON Biotechnology	**SAL200:** Bacteriophage endolysin	II Ongoing	NCT03089697	(178)
ContraFect	**CF-301:** Bacteriophage endolysin	II Completed	NCT03163556	(179)
Aridis	**Tosatoxumab:** Monoclonal antibody against Hla	III Recruiting	NCT03816956	(188)
AstraZeneca	**Suvratoxumab:** Monoclonal antibody against Hla	II Completed	NCT02296320	(189)
X-Biotech	**514G3:** Monoclonal antibody against SpA	II Completed	NCT02357966	(190)

Arsanis developed ASN-100, an antibody treatment consisting of two combined monoclonal antibodies that together target six lytic secreted toxins of *S. aureus*: Hla, PVL, gamma-hemolysin (HlgAB and HlgCB), LukED and LukGH (193, 194). ASN-100 was shown to protect rabbits during an experimental model of pneumonia to a much greater extent than protection offered by a Hla-neutralizing antibody alone (195). However, phase two clinical testing focusing on preventing the development of *S. aureus* VAP was halted before completion as it was deemed highly unlikely to meet its efficacy endpoints (NCT02940626).

Lastly, X-Biotech has developed 514G3, a monoclonal antibody against SpA, that unlike previously mentioned monoclonal antibody platforms, is focused on treating *S. aureus* bacteremia. In order to counteract SpA’s ability to sequester antibodies in their reverse orientation, 514G3 is an IgG3 isotype antibody, the sole subclass of IgG antibodies shown not to be bound in such a manner by SpA (196). Preclinical experiments demonstrated the efficacy of 514G3 in reducing *S. aureus* bacteremia-induced death in mice, while also showing an additive protective effect when mice where treated with vancomycin (197). 514G3 completed a combined phase I/II clinical trial on bacteremic patients in 2017 (190). Efficacy results from this trial have been publicly discussed by X-Biotech, who reported a reduction in *S. aureus* related adverse effects in the treated group vs a placebo group (11% *vs.* 26%) however, statistical significance was not reached. Similarly, a non-significant reduction was observed in the duration of hospital stay in bacteremic patients (p=0.092). Considering 36 patients received 514G3 and just 16 received placebo, it may be possible that significance could be achieved in a larger scale trial. It should also be noted that an investigation into whether or not treatment was associated with at least one death during the study was inconclusive, raising the possibility of safety concerns during future trials (https://investors.xbiotech.com/node/6796/pdf).

Overall, the use of monoclonal antibodies as therapeutic and short-term prophylactic treatments for *S. aureus* remains an area with indications of promise but requires further validation in larger controlled clinical studies. Interestingly, it has previously been hypothesized that treatment with monoclonal antibodies may result in an enhanced development of long-term immunity. It is proposed that antibody coating of pathogens or secreted antigens leads to the formation of immune complexes, which are readily phagocytosed by antigen presenting cells thereby driving the development of robust, protective anamnestic immune responses (198). This effect could be measured through post-treatment observation of patients looking specifically at *S. aureus* infection incidence.

### Further Experimental Treatments for *S. aureus*

Lesser studied but nonetheless highly promising strategies for treating *S. aureus* infections include centyrins, a novel class of therapeutic proteins based on the consensus sequence of the 15 fibronectin type 3 domains of the human protein Tenascin C (199). Centyrins can be considered as mimetics of monoclonal antibodies due to the fact that specific regions of the protein are highly mutable and may be selected for the ability to bind specifically to antigens with high affinity (199). Some notable differences when compared to monoclonal antibodies are that firstly, at less than 10% the size of IgG1 antibodies, centyrins are much smaller than monoclonal antibodies (200). Secondly, centyrins exist as unglycosylated single-chain proteins that lack disulfide bonds and therefore can easily be produced in large quantities in *E. coli*. Thirdly, due to the absence of an antibody Fc region, centyrins will not bind to cellular Fc receptors, thereby restricting their functionality to neutralization, not being able to induce opsonophagocytosis. Centyrins may therefore prove refractory to the sequestering effect of SpA on antibodies. Chan and colleagues recently developed centyrins capable of neutralizing the lytic activity of five bicomponent *S. aureus* toxins against human neutrophils, and further protected mice given otherwise lethal doses of LukED (201). The authors addressed the fact that centyrins have a short half-life which can be extended within human blood by fusing centyrins with albumin. This modification has previously been shown to extend centyrin half-life to approximately 7.5 days in Macaques, and yet, this is still far behind that of anti-toxin monoclonal antibodies used in previous clinical trials (21 – 112 days) (187, 192, 202).

The previously mentioned effect of trained immunity is an area that may prove interesting for the development of alternative short-term prophylactic strategies for individuals undergoing surgeries and therefore risking the acquisition of an infection. Such an idea is evidenced from older studies demonstrating the protective effect of prior *C. albicans* infection on a subsequent *S. aureus* infection (203), to more recent publications recapitulating the same effect *via* vaccination with fungal cell wall components (known inducers of trained immunity) (204). The power of this effect has been observed previously in children vaccinated with the Bacille Calmette-Guérin vaccine, another known inducer of trained immunity (205).

## Conclusion

Making a vaccine that can prevent *S. aureus* infection has proven to be challenging. *S. aureus* is a commensal organism of the human nasal mucosae and the skin that has adapted an array of armaments specifically focused on subverting the human immune system. In spite of this, the scientific community has continued to innovate and develop diverse and complex vaccine designs in order to evoke various arms of the immune system and to tackle many *S. aureus* virulence mechanisms. The bottleneck to licensure is the demonstration of efficacy at the clinical level. To give vaccines entering into clinical trials the best likelihood of success, validation at the preclinical level must be achieved using models that can better recapitulate the human condition during infection. Vaccines currently in clinical trials are using more defined endpoints, enrolling specific populations at risk of infection, targeting virulence and immune-evasion factors of *S. aureus* and have been shown to generate both humoral and cellular facets of the immune system.

The development of therapeutic and short-term prophylactic treatments for *S. aureus* infections is moving at an encouragingly high speed. Though in many ways the discussed treatments can be imagined as alternatives for vaccination, instead of running parallel to each other, these seemingly distinct fields can generate highly transferrable information and collaborative opportunity. For example, if proven to be efficacious in treating an infection, short-term immunotherapies may outline and inform the antigenic targets of *S. aureus* vaccines. Furthermore, considering that clinical trials will use standard of care therapies as a baseline for the therapeutic efficacy, understanding how immunotherapies, antibiotics and vaccines may synergize could be highly important in future clinical trial design. Our proposed future directions for *S. aureus* vaccinology research are listed in **Table 3**.

**Table 3:** Future directions of *Staphylococcus aureus* vaccinology research.

1. Developing and utilizing more representative models of infection	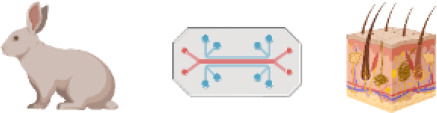
2. Understanding the consequences of pre-exposure	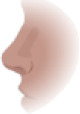
3. Determining the correlates of protection	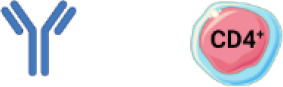
4. Designing clinical trials to capture vaccine efficacy	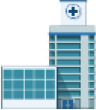
5. Disarming immune-evasion tactics employed by *S. aureus*	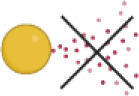

## Author Contributions

JC wrote the manuscript. JC, ES, SR, FB, RM, and SP revised and approved the manuscript. All authors contributed to the article and approved the submitted version.

## Funding

This work was co-sponsored by GlaxoSmithKline Biologicals SA and associated research in Trinity College Dublin was supported by a Science Foundation Ireland Investigator Award (15/IA/3041) to RM. The funders was not involved in the study design, collection, analysis, interpretation of data, the writing of this article or the decision to submit it for publication.

## Conflict of Interest

JC is a PhD fellow who is enrolled in the School of Biochemistry and Immunology at Trinity College Dublin and participates in a postgraduate studentship program at GSK. ES, SR, FB, and SP are employees of the GSK group of companies.

The remaining author declares that the research was conducted in the absence of any commercial or financial relationships that could be construed as a potential conflict of interest.
